# QTL Mapping of Heat Tolerance in Cucumber (*Cucumis sativus* L.) at Adult Stage

**DOI:** 10.3390/plants10020324

**Published:** 2021-02-08

**Authors:** Yanyan Liu, Shaoyun Dong, Shuang Wei, Weiping Wang, Han Miao, Kailiang Bo, Xingfang Gu, Shengping Zhang

**Affiliations:** Institute of Vegetables and Flowers, Chinese Academy of Agricultural Sciences, Beijing 100081, China; 82101182238@caas.cn (Y.L.); dongshaoyun@caas.cn (S.D.); 82101176052@caas.cn (S.W.); weiping.wang@pku-iaas.edu.cn (W.W.); miaohan@caas.cn (H.M.); bokailiang@caas.cn (K.B.)

**Keywords:** cucumber (*Cucumis sativus* L.), heat tolerance, genetic analysis, QTL mapping, candidate gene analysis

## Abstract

Heat stress during cucumber production often leads to sunburn of leaves, growth retardation of stems and roots, fruit malformation, and even plant death, which have a great impact on the fruit quality and yield. However, no studies on the genetic inheritance and quantitative trait locus mapping of heat tolerance in cucumber at the adult stage have been reported yet. In this study, a set of 86 recombinant inbred lines (RILs) derived from “99281” (heat-tolerant) and “931” (heat-sensitive) were used to identify the heat tolerance QTL in summer 2018, 2019, and 2020. Eight-week-old plants were exposed to a natural high temperature environment in the field, and the heat injury index was used to indicate the heat tolerance performance. Genetic analysis showed that the heat tolerance of adult cucumber is quantitatively inherited. One QTL named *qHT1.1* on chromosome 1 was identified. It was delimited by Indel 3-3 and Indel 1-15 and explained 59.6%, 58.1%, and 40.1% of the phenotypic variation in 2018, 2019, and 2020, respectively. The efficiency of marker HT-1, which is closely linked to the locus, was tested using 62 cucumber germplasm accessions and was found to have an accuracy of 97.8% in heat sensitive plants. The *qHT1.1* was delimited to a 694.5-kb region, containing 98 genes, nine of which may be involved in heat tolerance. Further sequence analysis showed that there are three single-base substitutions within the coding sequences of *Csa1G004990*. Gene expression analyses suggested that the expression of *Csa1G004990* was significantly higher in “99281” than “931” at 14d, 35d, 42d, and 49d after transplanting. This study provides practically useful markers for heat tolerance breeding in cucumber and provides a basis for further identifying heat tolerant genes.

## 1. Introduction

Cucumber (*Cucumis sativus* L.) is one of the most important vegetable crops worldwide. Global production of cucumber reached 75.2 million metric tons in 2019, 74.8% of which was from China (http://www.fao.org/faostat/en/#data/QC/visualize (accessed on 15 February 2020)). Cucumber originated from tropical areas but is sensitive to high temperature [1]. The optimal temperature for its growth is 25–28 °C during the day and 15–20 °C at night [2]. With the increasing global temperature, cucumber is more susceptible to heat stress (HS) in production, especially in the late spring and early autumn of facility cultivation, where the temperature often exceeds 35 °C or even 50 °C [2]. In summer, the temperature of cultivation in open fields often exceeds 35 °C, which leads to sunburn of leaves, growth retardation of stems and roots, fruit malformation, and even plant death, which severely affect cucumber yield and fruit quality [3,4].

In previous studies, the heat tolerance of cucumber was mainly evaluated at the seedlings stage. The electrical conductivity was the most widely used heat tolerance indicator [5,6,7,8]. The content of superoxide dismutase [5,9], and the heat damage calculated based on the dryness area of leaves [10,11] were also be used to indicate the heat tolerance performance at the seedling stage. However, few studies on the evaluation of the heat tolerance in adult cucumber have been reported yet, and there is no uniform standard. Meng and Zhang [6,12] took the yield loss as the index of heat tolerance ability, and other traits including plant height, stem diameter, leaf area, and number of female flowers on main stem were also used to determine the heat tolerance of adult cucumber [13,14].

Most previous studies suggested that the heat tolerance in cucumber seedlings is a quantitative trait that is controlled by multiple genes; however, no genetic studies at adult stage has been reported yet. Using heat-tolerant line “R1” and heat-sensitive line “R29”, it was found that heat tolerance fits an additive–dominant model, mainly with an additive effect [15]. Zhang [12] found that heat tolerance was determined by major genes with an additive effect. Li [16] took the heat injury index as the indicator and found that the heat tolerance of cucumber at the seedling stage was controlled by two major genes and multiple minor genes. Zhuang found that heat tolerance of cucumber seedling complied with the E-1 model, which was controlled by additive–dominance and additive–additive interaction of two major genes and an additive effect of multiple genes [17]. Xu et al. [18] reported that the heat tolerance of cucumber seedling was controlled by two major genes with an additive–dominant–epistasis effect and polygene with an additive–dominant effect. Recently, Wang et al. [19] showed that the heat tolerance of “L-9” (heat-resistant) seedling was controlled by a single recessive gene. Different conclusions in these studies might due to different experimental materials, heat stress treatment, and evaluation standard.

Few studies on QTL/gene mapping of heat tolerance in cucumber at the seedling stage have been reported. Yang [20] identified one simple sequence repeat (SSR) marker linked to the heat tolerance of cucumber seedlings using the heat-resistant inbred line “Poinsett 97” and the heat-sensitive inbred line “Boothbyls Blonde”. Yang used relative electrical conductivity as an index and identified four SSR and seven random amplified polymorphic DNA (RAPD) markers related to heat tolerance [21]. Zhuang identified one QTL on chromosome 5 with the phenotypic variation rate of 11% [18]. Wang et al. [19] showed that the heat tolerance of cucumber seedlings in “L-9” (heat-resistant) was controlled by a single gene on chromosome 1, flanked by Indel-H90 and Indel-H224. Dong et al. [22] identified six QTLs including *qHT3.1*, *qHT3.2*, *qHT3.3*, *qHT4.1*, *qHT4.2*, and *qHT6.1*; among them, the phenotypic interpretation rate of *qHT3.2* was 28.3%. However, no molecular markers that are closely linked to a heat tolerance gene have been developed, and no genes have been cloned in these studies. In addition, no genes related to heat tolerance at the adult stage have been mapped yet.

Plants activate a variety of events in response to heat stress, including the regulation of antioxidation protein activity, positive regulation in response to HS, and hormone signaling responses [23]. Studied have showed that JMJC domain protein can respond to HS by affecting DNA methylation [24]. MYB family transcription factor is widely involved in various physiological processes and responds to heat stress [25]. WD-repeat protein may interact with MYB transcription factor in response to HS [26]. Moreover, zinc protein has a positive regulatory effect on HS [27]. According the report, overexpression of the calmodulin gene, namely *CsCaM3*, prevents peroxidation and photosynthesis system damage in response to heat stress [28].

The objective of this study was to identify QTLs for heat tolerance in cucumber at the adult stage using a recombinant inbred lines (RIL) population constructed from the heat tolerance line “99281” and heat sensitive line “931” and to analyze candidate genes. The results from this study promote the breeding of heat tolerance cucumber varieties and provide a basis for further fine mapping.

## 2. Results

### 2.1. Inheritance Analysis of Heat Tolerance in Adult Cucumber

**Figure 1:**
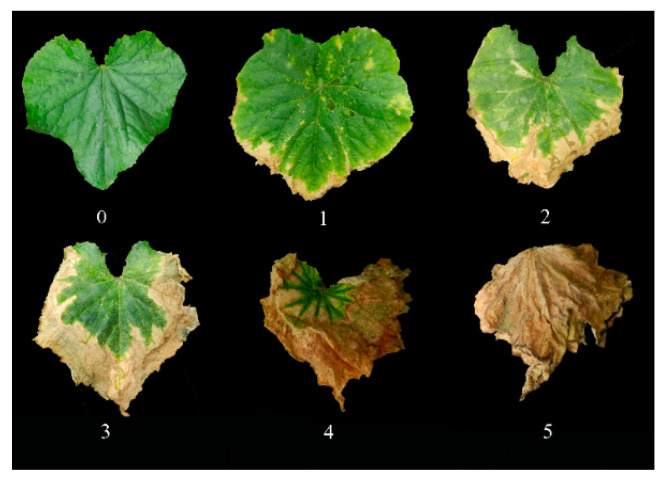
Phenotypic characterization of heat tolerance in cucumber adult plant: 0 = no symptoms on the 8th to 10th leaves; 1 = only edges of the 8th to 10th leaves were dried; 2 = 1/3 of the 8th to 10th leaves were dried; 3 = 1/2 of the 8th to 10th leaves were dried; 4 = more than 2/3 of the 8th to 10th leaves were dried; 5 = all of the 8th to 10th leaves were dried.

To analyze the inheritance pattern of heat tolerance in cucumber adult plant, the parental lines, F_1_, and RIL population were grown in the open field at two locations over three years. In 2018–2020, eight-week-old plants were exposed to natural heat stress (0.5–6.5 h of daily temperature above 35 °C) for 17 days at Shunyi, Beijing (SY) and 18 days at Nankou, Beijing (NK), respectively. Symptoms of heat stress became obvious at 42 days after transplanting. After the stress treatment, the heat injury was classified into six grades, based on the overall dryness area of the 8th to 10th leaves (Figure 1). The heat injury index (HII) was used to indicate the heat tolerance performance of each plant.

**Figure 2:**
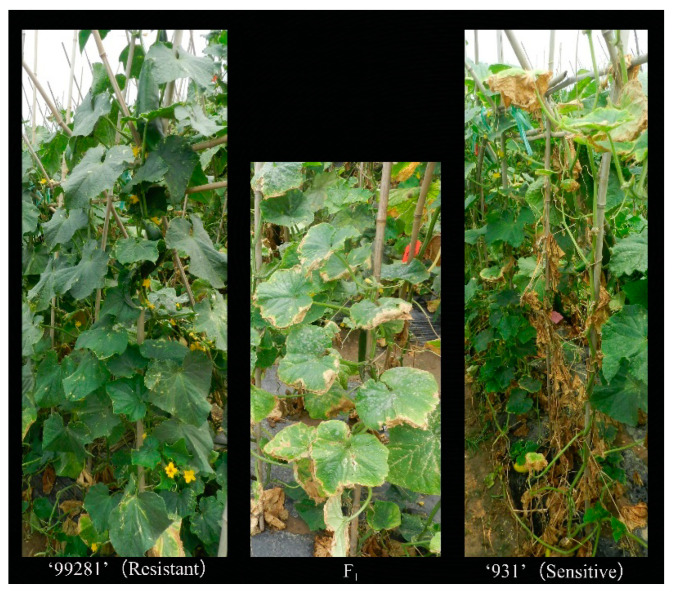
Phenotypes of parents and F_1_ under heat stress. Parental line “99281” is heat-tolerant with no obvious damage on the leaves, and “931” is heat-sensitive with leaves wilting and the whole plant died. F_1_ showed moderate heat tolerance.

**Figure 3:**
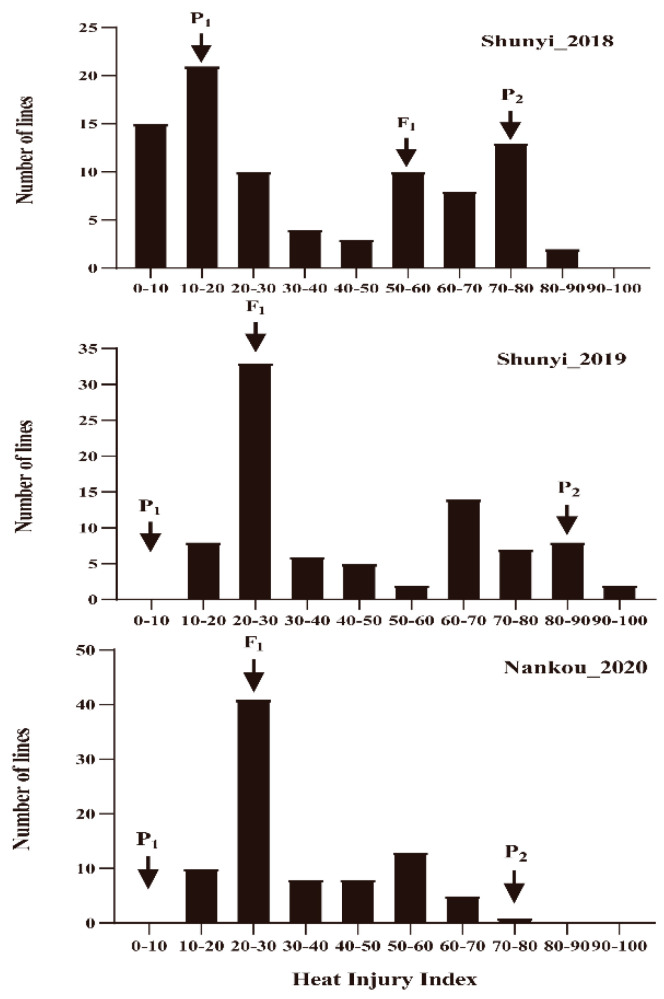
Frequency distribution of heat injury index in RIL populations in 2018, 2019, and 2020. *X*-axis indicates the heat injury index (HII); *Y*-axis indicates the number of individuals in each HII category. P_1_: parental line “99281” (heat-tolerant) and P_2_: parental line “931” (heat-sensitive). Arrows indicate the average HII of parental and F_1_ lines.

Results suggested that “99281” and “931” showed strong tolerance and sensitivity to heat stress, respectively, in three environments. Parental line “99281” grew normally, and the 8th to 10th leaves showed no significant damage, with HIIs of 17.50, 14.00, and 9.33 in 2018, 2019, and 2020, respectively. However, parental line “931” was completely dead, with HIIs of 86.50, 93.75, and 73.33, respectively. The HIIs of F1 was 50.00, 24.00, and 26.00 in 2018, 2019, and 2020, with the performance more inclined to “99281” (Figure 2, Table 1). The HII of the RIL population followed a continuous variation from tolerance to sensitive phenotypes (Figure 3), suggesting that resistance heat tolerance in cucumber at adult stage was controlled by multiple genes.

### 2.2. QTL Mapping of Heat Tolerance

**Figure 4:**
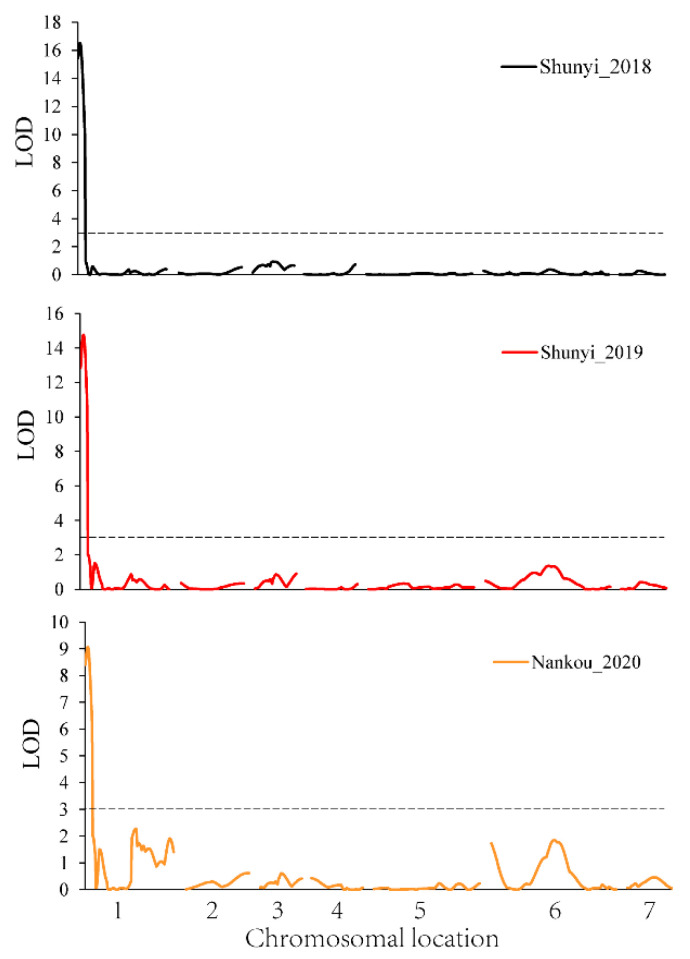
QTL mapping of heat tolerance in cucumber at adult stage. *X*-axis indicates the genetic position of each chromosome; *Y*-axis indicates the LOD value. The black, red, and orange lines indicate experiments conducted at Shunyi_2018, Shunyi_2019, and Nankou_2020. One QTL on chromosome 1 was repeatedly detected in 2018, 2019, and 2020.

**Figure 5:**
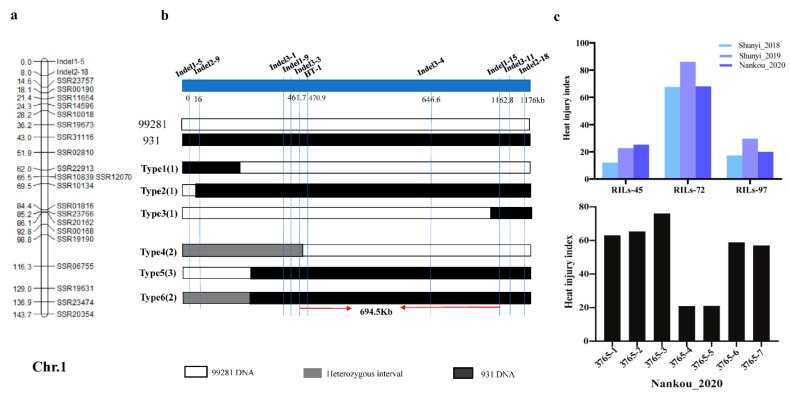
Fine mapping of *qHT1.1*. (**a**) QTL mapping of *qHT1.1* in 2018, 2019, and 2020. (**b**) A total of 10 Indel markers were used to fine map the *qHT1.1* to a 694.5-Kb region on Chr.1. (**c**) The phenotypic data of RIL population and F_2_ population. Type1, Type2, and Type3 represent RIL-45, RIL-72, and RIL-97, respectively. Type4 represents 3765-4 and 3765-5. Type5 represents 3765-1, 3765-2, and 3765-3. Type6 represents 3765-6 and 3765-7.

We previously developed a linkage map for the “99281” × “931” RIL population, which contains 78 SSR markers, spanning 521.3 cM, with an average marker interval of 6.68 cM [29]. Combing the genetic map and phenotypic data, one QTL on chromosome 1, named *qHT1.1*, was repeatedly detected in 2018, 2019, and 2020. The logarithm of odds (LOD) scores in 2018, 2019, and 2020 were 20.96, 24.84, and 12.72, respectively, and accounted for 59.60%, 58.10%, and 40.10% of the phenotypic variation, respectively (Figure 4). Details of QTL detected, including chromosome number, marker interval, peak location, logarithm of odds (LOD) support value, and percentages of total phenotypic variances explained (R^2^), are shown in Table 2. The flanking marker SSR23757 is 1.9 Mb from *qHT1.1*. To refine the preliminary mapping region, 10 Indel markers (Supplementary Table S4) were designed based on the sequence information of “99281” and “931”. Additionally, *qHT1.1* was further mapped between the markers Indel 2-9 and Indel 1-15. Indel 1-5 and Indel 2-18 were selected to screen the 500 F_2_ plants, and seven recombinants were obtained (Table S3) and further mapped the qHT1.1 between the markers Indel 3-3 and Indel 1-15, with the physical distance of 694.5 Kb (702,518–1,396,993 bp) (Figure 5).

### 2.3. Validation of Molecular Marker Linked to qHT1.1

**Figure 6:**
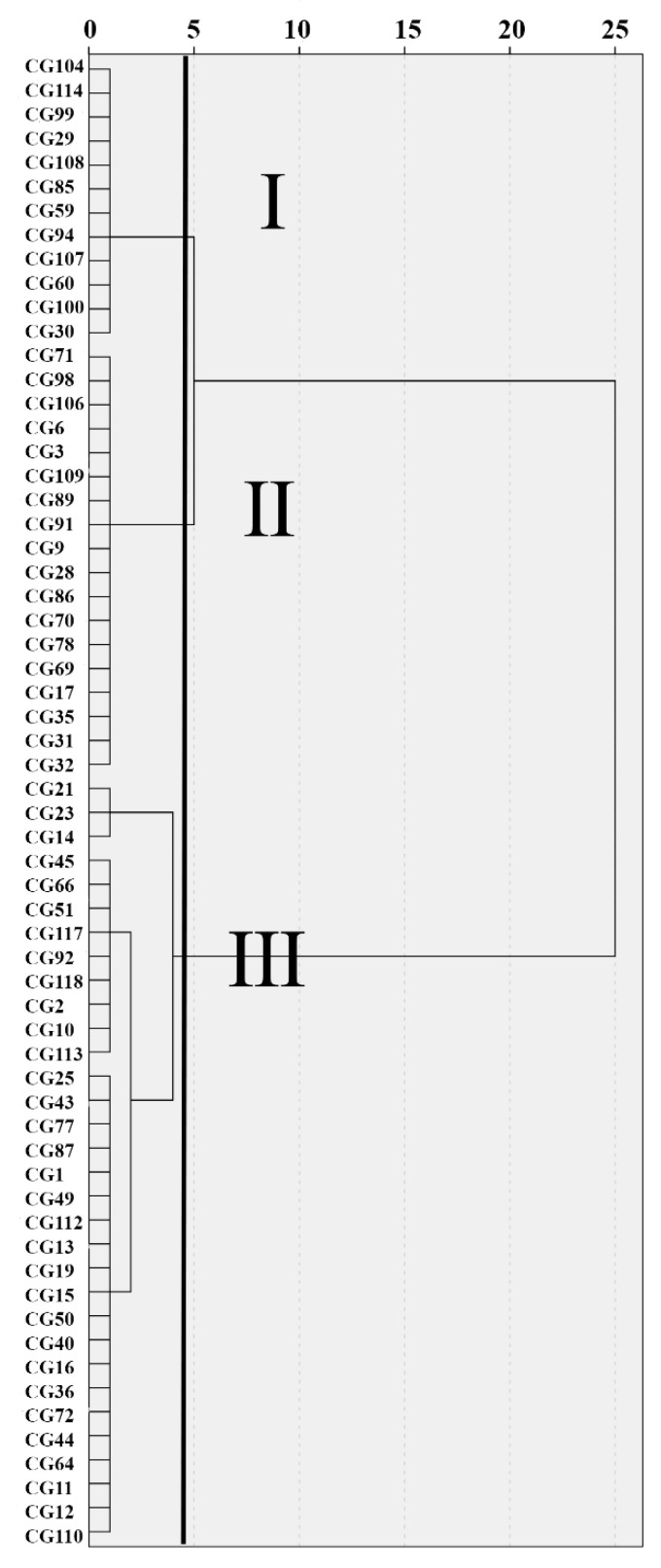
Cluster analysis of 62 germplasm accessions based on HII value. The line with HII less than 35 belongs to group Ⅰ, the line with HII greater than 35 and less than 50 belongs to group Ⅱ, and the line with HII greater than 50 belongs to group Ⅲ.

HT-1, the closest marker linked to *qHT1.1*, was tested on 62 core germplasm (CG) lines. Firstly, cluster analysis was carried out based on the HII of CG, and the result showed that 62 individuals were categorized into three groups. The line with HII less than 35 belonged to group Ⅰ; the line with HII greater than 35 and less than 50 belonged to group Ⅱ; and the line with HII greater than 50 belonged to group Ⅲ (Figure 6). Secondly, the result of CG genotype identification using HT-1 marker was combined. The final analysis shows that 16 materials were clustered to Ⅰ, 14 materials were clustered to Ⅱ, and 32 materials were clustered to Ⅲ. For one line that was either heat sensitive or moderate (2), the genotype did not match the phenotype. For six of the heat resistant lines (32, 33, 42, 50, 54, and 60), the genotype and phenotype were inconsistent (Supplementary Table S5). Thus, marker HT-1 was 97.8% accurate in heat sensitive or moderate lines and 62.5% accurate in heat resistant lines. The marker HT-1 can be used in molecular marker-assisted breeding.

### 2.4. Prediction of HT Candidate Gene

**Figure 7:**
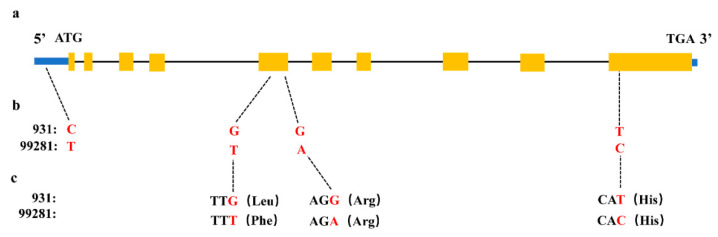
Nucleic acid and amino acid changes of *Csa1G004990*. (**a**) Gene structure of *Csa1G004990*. (**b**) One single-nucleotide substitutions (C/T) in the 5′-untranslated region. Two single-nucleotide substitution (G/T, G/A) in 5^th^ exon and one single-nucleotide substitutions (T/C) in 10th exon. (**c**) Among the three single-nucleotide substitution, one caused amino acid substitution (Leu/Phe). The left blue box indicates the 5′-untranslated region, the orange box indicates coding regions, the black line indicates an intron, and the right blue box indicates the 3′-untranslated region.

The QTL was delimited to a 694.5-Kb region (702,518–1,396,993 bp) between Indel 3-3 and Indel 1-15, harboring 98 genes (listed in Supplementary Table S6) according to the gene annotation from the Cucurbit Genomics Database (http://cucurbitgenomics.org/ (accessed on 21 October 2020)). According to previous reports, JMJC protein, zinc protein, U-box protein, calmodulin protein, and MYB family transcription factor are related to heat tolerance [7,25,27,30,31,32]. Among these 98 genes, nine were found to be related to heat stress response; the predicted function and associated information of these nine candidate genes are listed in Table 3. They are genes encoding zinc protein (*Csa1G004350*, *Csa1G004990*, *Csa1G008480*) that are positively involved in HS, antioxidant proteins like JMJC protein, WD-repeat protein and U-box protein (*Csa1G004270*, *Csa1G004880*, *Csa1G005730*), calmodulin protein (*Csa1G005690*), and two MYB transcription factors. Based on the resequencing data, we examined polymorphisms of these nine genes between “99281” and “931” and found that *Csa1G004990* had three single-base substitutions within the coding sequences (CDSs) and one single-base substitutions in its 5′-untranslated region; however, the other eight genes showed 100% nucleotide sequence identity in their exons. *Csa1G004990* had ten exons and nine introns, all of which encoded a predicted protein of 244 amino acids (Figure 7a). The single base substitutions caused an amino acid substitution in exon 5, and only one single base substitution at position 1552 caused a change at the 94th amino acid (Figure 7b,c). The functional annotation of *Csa1G004990* revealed that the gene encodes a ring finger protein.

### 2.5. Gene Expression Pattern Analysis

**Figure 8:**
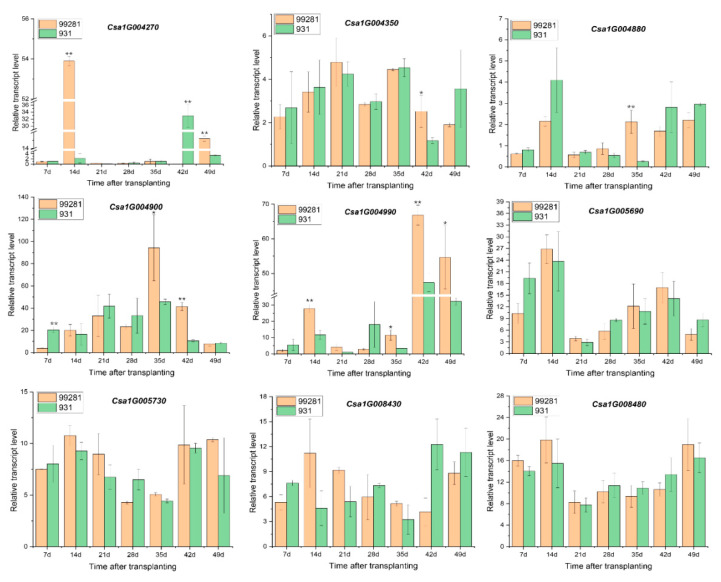
Relative quantitative expression analysis of candidate genes. The data represent the relative expression levels of genes at different time-points in “99281” and “931”. Actin1 was set as the internal reference gene. The error bars represent the standard error of three biological replicates. The seven time-points before (7d, 14d, 21d, 28d, 35d after transplanting) and after (42d and 49d after transplanting) heat stress of “99281” and “931” correspond to the seven columns in the histogram. The asterisks indicate that there were significant differences in expression level between “99281” and “931” (* 0.01 < *p* < 0.05; ** *p* < 0.01).

The temporal expression pattern of the nine candidate genes that before (at 7d, 14d, 21d, 28d, 35d after transplanting) and after (at 42d and 49d after transplanting) heat stress was examined in “99281” and “931” (Table 3). The result showed that the expression of *Csa1G004990* in “99281” was significantly higher than “931” at 14d, 35d, 42d, and 49d after transplanting, and the expression level of this gene was increased from 7d to 49d after transplanting in “99281”. The expression level of *Csa1G004270* in “99281” was significantly higher than “931” at 14d and 49d, but reversed at 42d. Similarly, the expression level of *Csa1G004900* in “99281” was higher than “931” at 35d and 42d, but reversed at 7d. The expression levels of *Csa1G004350* and *Csa1G004880* in “99281” were higher than “931” in 42d and 35d, respectively. The expression levels of *Csa1G005690*, *Csa1G005730*, *Csa1G008430*, and *Csa1G008480* had no significant differences between “99281” and “931” (Figure 8). Therefore, *Csa1G004990* may be involved in the regulation of heat tolerance in cucumber adult plant.

## 3. Discussion

There are few reports on the evaluation of heat tolerance in adult cucumber so far. Previous studies used plant height, stem diameter, leaf area, female flower number [13,14], and yield loss [6,12] as the indexes to indicate the heat tolerance performance in cucumber adult plant. In this study, we referred to the evaluation standard of leaf damage at seedling stage and used the overall dryness area of 8th–10th leaves to indicate the heat tolerance ability in adult plant, which makes it easier to compare the heat tolerance at different stage. Moreover, this study was carried out in the open field in two locations over three years, which is similar to the natural high temperature conditions that cucumber suffered in production. Therefore, the results can be directly applied for solving problems in cucumber production.

At present, studies on the heat tolerance of cucumber were mostly carried out at the seedling stage. It was reported that the heat tolerance of cucumber seedling was controlled by multiple genes [15,16,17,18] or a single gene [19]. The different conclusions may due to the different experimental materials, treatment conditions, and evaluation criteria. In this study, we analyzed the inheritance of heat tolerance in adult cucumber using RIL populations generated from inbred lines “99281” (heat resistant) and “931′ (heat sensitive) and found that heat tolerance in “99281” adult plant was controlled by a major QTL. It was the first time that the inheritance pattern of heat tolerance was reported at the adult stage in cucumber.

Few QTL/genes for heat tolerance in cucumber seedlings have been identified so far. Zhuang [17] identified one QTL on chromosome 5 using the heat injury index as an indicator, and the phenotypic variation rate was 11%. Wang et al. [19] found that the heat tolerance of cucumber seedling was controlled by a single gene, and they mapped the gene to a 550-kb region on chromosome 1, flanked by Indel-H90 and Indel-H224 (20,060,447–20,585,920 bp). In our study, the *qHT1.1* in adult plant of “99281” was mapped on chromosome 1 defined by Indel markers Indel 3-3 and Indel 1-15 (702,518–1,396,993bp), which is 18.7 Mb away from the region mapped in Wang et al. [20]. Therefore, heat tolerance in cucumber at seedling and adult stages may be determined by different genes. Compared with reported QTLs for heat tolerance, the *qHT1.1* identified in our study is easier for further gene cloning and application for breeding.

The physical length of the qHT1.1 region was 694.5 kb (702,518–1,396,993 bp), harboring 98 genes. Within this region, *Csa1G004270* that encodes JMJC domain protein, *Csa1G004880* that encodes WD-repeat protein, and *Csa1G005730* that encodes U-box protein were each identified. Studies have shown that JMJC domain protein can respond to heat stress by affecting DNA methylation [24]. WD-repeat protein may interact with MYB transcription factor in response to HS [26]. The U-box protein may respond to HS as a molecular chaperone [31]. Moreover, *Csa1G005690*, which encodes a calmodulin protein, was also found within this region. A previous study suggested that overexpressing *CsCaM3*, a calmodulin gene, could prevent peroxidation and photosynthesis system damage and thus improve heat tolerance [28]. Two transcription factors, including *Csa1G004900* and *Csa1G008430,* which encode an MYB transcription factor, were also found in the QTL. Transcription factors play an essential role in how plants respond to abiotic stress through signal-linked transduction [33]. *Csa1G004350*, *Csa1G004990*, and *Csa1G008480* encode CW-type zinc finger protein, RING finger protein, and CHY zinc finger domain-containing protein, respectively. Zinc protein has a positive regulatory effect on HS [27]. In our study, sequence analysis of *Csa1G004990* between “99281” and “931” showed that *Csa1G004990* had three single-base substitutions within the coding sequences. Moreover, *Csa1G004990* is significantly up-regulated by HS. However, if and how these genes are involved in heat tolerance need to be further studied.

## 4. Materials and Methods

### 4.1. Plant Materials

The parental “99281”, a northern Europe greenhouse type, is an inbred line that is tolerant to heat stress, and “931” is a Northern China fresh market type with heat sensitivity (Figure 2). For QTL mapping of heat tolerance in adult cucumber, a population of 86 RILs and F_2_ developed from a cross between “931” and “99281” were used. The RIL population was developed by single seed descent. The F_2_ population, which contained 500 individuals, was employed for fine mapping of the *qHT1.1*. All materials were preserved by the Cucumber Research Group, Institute of Vegetables and Flowers (IVF), Chinese Academy of Agricultural Sciences (CAAS).

### 4.2. Phenotypic Analysis of Heat Tolerance

Three experiments were conducted to evaluate the heat tolerance of the parental lines, F1 and RIL population in summer 2018, 2019, and 2020, respectively. Plants were grown in the open field at Shunyi (SY), Beijing, China (40°8’ N; 116°52’ E; altitude 38 m) in 2018 and 2019 and Nankou (NK), Beijing, China (40°13’ N; 116°14’ E; altitude 53 m) in 2020. The experiment was designed using a completely randomized block design method with three replicates in each year and five plants for each replicate. In 2018–2020, eight-week-old plants were exposed to a natural high temperature environment, and the symptom of heat stress become obvious at 42 days after transplanting. In 2018–2020, temperatures were recorded for 20 days; the detailed real-time temperature during the heat stress treatment is listed in Supplementary Materials Table S1. After heat stress treatment, phenotypic data of the heat injury in RILs population were collected, and the heat injury was classified into six grades according to the average dryness area of three (the 8th to 10th) entire leaves (Figure 1). The index used was as follows: 0 = no damage on the 8th to 10th leaves; 1 = only edges of the 8th to 10th leaves were dried; 2 = 1/3 of the 8^th^ to 10th leaves were dried; 3 = 1/2 of the 8th to 10th leaves were dried; 4 = more than 2/3 of the 8th to 10th leaves were dried; 5 = the entire 8th to 10th leaves were dried. Then the heat injury index (HII) was calculated to present the heat tolerance performance of each plant as previously reported [34] using the formula as follows: HII = ((0 × S0 + 1 × S1 + 2 × S2 + 3 × S3 + 4 × S4 + 5 × S5)/(5 × N)) × 100. S0–S5 indicates the number of plants corresponding to each grade. N indicates the total number of plants. For each experiment, the HII of each line was calculated by taking the average of the HII in three replicates.

### 4.3. QTL Mapping of Heat Tolerance

In our previous study, a linkage map for the RIL population (“99281” × “931”) was developed with 78 SSR [29]. Phenotypic data of heat tolerance in 2018, 2019, and 2020 (Supplementary Table S2) were employed in the QTL mapping analysis. The QTL software package in R (R/QTL) was used for QTL mapping with LOD > 3.0 (http://www.rqtl.org/ (accessed on 15 September 2020)). The detected locus was named in accordance with the following scheme: the abbreviation of HT, chromosome (Chr.) number, and locus number [35]. Since the preliminary mapping region was still large, polymorphic Indel markers were used to narrow down the region. The Indels were identified by analyzing the genomic data of two parental lines. Primers were designed with Primer3web (http://bioinfo.ut.ee/primer3-0.4.0/ (accessed on 15 September 2020)) following Gao et al. [36]. To narrow down the region of major QTL, at least 20 plants of each F_2_-derived F_3_ population were scored to infer the phenotype at the *qHT1.1* in each F_2_ recombinant plant. The method of characterizing heat tolerance was the same as that described in Section 2.2. According to the results of preliminary mapping, Indel 1–5 and Indel 2–18 on chromosome 1 were used as flanking markers to select the recombinant individual line in F_2_ population (Supplementary Table S3).

### 4.4. Validation of Indel Markers Linked to Heat Tolerant

The accuracy of markers that were closely linked to heat tolerant was evaluated on 62 CG from different geographical origins. The detailed geographical origin, heat tolerance phenotype, heat injury index, and genotype of the CG lines are listed in Supplementary Table S5. The method of characterizing heat tolerance is the same as described in Section 4.2. Marker HT-1 is a newly developed marker, which is closely linked with *qHT1.1*, and its sequence information is shown in the Table S4. It was used to identify genotype of 62 materials. For each individual, the genotypes consistent with “99281” were categorized as “a”, and those consistent with “931” were categorized as “b”. The cluster analysis of 62 materials was carried out based on the calculated HII.

### 4.5. Prediction of Candidate Gene

The prediction of the candidate gene was based on the gene annotation in the reference genome of cucumber “Chinese long V2.0” (http://cucurbitgenomics.org/organism/2 (accessed on 23 December 2020)). Genes associated with stress resistance such as JMJC domain protein [24], zinc protein family [28], calmodulin protein [31], U-box protein [31], and MYB family transcription [25,34] were selected. Based on the resequencing data of “99281” (R374) and “931” (CG1601) [37], polymorphisms of the selected genes between “99281” and “931” were examined. The sequence data of “R374” was unpublished, and the sequence data of “CG1601” was extracted from the National Center for Biotechnology Information Short Read Archive (SRA) under accession SRA056480.

### 4.6. Extraction of RNA and qRT-PCR

Cucumber leaves of “99281” and “931”were collected before (at 7d, 14d, 21d, 28d, 35d after transplanting) and after (at 42d and 49d after transplanting) heat stress, respectively. The total RNA was extracted using an RNeasy Plant Mini Kit (TaKaRa 9769, Takara Bio, Inc. Otsu, Japan). The qRT-PCR was conducted using SYBR Premix Ex TaqTM II (TaKaRa, Takara Bio, Inc., Otsu, Japan). Actin1 was used as a reference gene for normalizing gene expression values [38]. Specific primers for each gene are listed in Supplementary Table S7. Three biological replicates were set for each treatment, and three technical replicates were performed. Relative gene expressions were calculated by using the 2^−∆∆Ct^ method [39].

### 4.7. Statistical Analysis

The significant differences of heat injury index and candidate gene expression level between “99281” and “931” were analyzed using one-way ANOVA in the IBM SPSS environment. GraphPad Prism 8 [40] was used for chart preparation.

## 5. Conclusions

In this study, we reported that heat tolerance in cucumber at the adult stage is controlled by multiple genes and is a quantitative trait. The loci, namely *qHT1.1*, was repeatedly detected in three years. Furthermore, candidate genes within this QTL that are involved in heat stress response were predicted. This study lays a foundation for further study of heat tolerance in cucumber at the adult stage and provides an idea for research of molecular mechanisms of cucumber heat tolerance.

## Figures and Tables

**Table 1:** The heat injury index of the parental lines, F_1_ plants, and recombinant inbred lines (RIL) populations.

Environment	Parental Lines (Mean ± SE)		F1 (Mean ± SE)	Homozygous Individuals of RILs			
	“99281”	“931”		(Mean ± SE)	SD	Skewness	Kurtosis
Shunyi_2018	17.50 ± 2.50 **	86.50 ± 1.50	50.00 ± 2.00	36.43 ± 2.88	26.75	0.39	−1.44
Shunyi_2019	14.00 ± 2.00 **	93.75 ± 1.03	24.00 ± 1.79	44.85 ± 2.69	24.96	0.48	−1.24
Nankou_2020	9.33 ± 1.33 **	73.33 ± 0.00	26.00 ± 2.00	32.62 ± 1.67	15.52	0.80	−0.36

Note: ** indicates that the value is significant at *p* ≤ 0.01.

**Table 2:** QTL controlling heat tolerance in adult cucumber.

Environment	Population	QTL	Chromosome	Marker Interval	Peak Location	LOD	R^2^ (%)
Shunyi_2018	RIL	*qHT1.1*	Chr.1	SSR23757	4.0 cM	20.96	59.60
Shunyi_2019	RIL	*qHT1.1*	Chr.1	SSR23757	4.0 cM	24.84	58.10
Nankou_2020	RIL	*qHT1.1*	Chr.1	SSR23757	4.0 cM	12.72	40.10

**Table 3:** Analysis of candidate genes related to cucumber heat tolerance.

Gene ID	Location on Chr.1	Gene Function Annotation
*Csa1G004270*	757,308–761,377	JmjC domain-containing protein
*Csa1G004350*	807,495–821,790	MORC family CW-type zinc finger protein
*Csa1G004880*	848,104–850,314	WD repeat-containing protein
*Csa1G004900*	858,938–861,275	Myb family transcription factor
*Csa1G004990*	987,005–990,966	RING finger protein
*Csa1G005690*	1,120,999–1,126,651	Calmodulin binding protein
*Csa1G005730*	1,142,413–1,144,240	U-box domain-containing protein
*Csa1G008430*	1,275,027–1,276,369	MYB transcription factor
*Csa1G008480*	1,297,918–1,307,476	RING finger and CHY zinc finger domain-containing protein

## Data Availability

Data are contained within the article or Supplementary Material.

## Supplementary Materials

The following are available online at https://www.mdpi.com/2223-7747/10/2/324/s1: Table S1. Heat stress treatment conducted at Shunyi and Nankou in 2018, 2019, and 2020; Table S2. Phenotypic data of heat tolerance in 2018, 2019, and 2020; Table S3. Heat tolerance phenotypic data of F_2_ population; Table S4. Oligos used for gene mapping in this study; Table S5. Validity of the marker HT-1 tightly linked with HT in 62 core germplasm materials; Table S6. Analysis of candidate genes related to cucumber heat stress; Table S7. Oligos used for the real-time quantitative PCR.

## Abbreviations

QTL	quantitative trait loci
RIL	recombinant inbred line
HS	heat stress
HII	heat injury index
SY	Shunyi
NK	Nankou
CG	core germplasm
SSR	simple sequence repeat
RAPD	random amplified polymorphic DNA
R/QTL	QTL software package in R
